# Timed Bromocriptine-QR Therapy Reduces Progression of Cardiovascular Disease and Dysglycemia in Subjects with Well-Controlled Type 2 Diabetes Mellitus

**DOI:** 10.1155/2015/157698

**Published:** 2015-04-28

**Authors:** Bindu Chamarthi, J. Michael Gaziano, Lawrence Blonde, Aaron Vinik, Richard E. Scranton, Michael Ezrokhi, Dean Rutty, Anthony H. Cincotta

**Affiliations:** ^1^Division of Endocrinology, Diabetes and Hypertension, Brigham and Women's Hospital, 221 Longwood Avenue, Boston, MA, USA; ^2^Harvard Medical School, Boston, MA, USA; ^3^VeroScience LLC, 1334 Main Road, Tiverton, RI 02878, USA; ^4^Veterans Affairs Healthcare System, 1400 VFW Parkway, W Roxbury, Boston, MA 02132, USA; ^5^Divisions of Aging, Cardiology and Preventive Medicine, Brigham and Women's Hospital, 75 Francis Street, Boston, MA, USA; ^6^Ochsner Medical Center, 1514 Jefferson Hwy, New Orleans, LA 70121, USA; ^7^Eastern Virginia Medical School Strelitz Diabetes Center and Neuroendocrine Unit, 855 W. Brambleton Avenue, Norfolk, VA 23510, USA; ^8^Everest Clinical Research Services Inc., 675 Cochrane Dr., Markham, ON, Canada L3R 0B8

## Abstract

*Background. * Type 2 diabetes (T2DM) patients, including those in good glycemic control, have an increased risk of cardiovascular disease (CVD). Maintaining good glycemic control may reduce long-term CVD risk. However, other risk factors such as elevated vascular sympathetic tone and/or endothelial dysfunction may be stronger potentiators of CVD. This study evaluated the impact of bromocriptine-QR, a sympatholytic dopamine D2 receptor agonist, on progression of metabolic disease and CVD in T2DM subjects in good glycemic control (HbA1c ≤7.0%).* Methods.* 1834 subjects (1219 bromocriptine-QR; 615 placebo) with baseline HbA1c ≤7.0% derived from the Cycloset Safety Trial (this trial is registered with ClinicalTrials.gov Identifier: NCT00377676), a 12-month, randomized, multicenter, placebo-controlled, double-blind study in T2DM, were evaluated. Treatment impact upon a prespecified composite CVD endpoint (first myocardial infarction, stroke, coronary revascularization, or hospitalization for angina/congestive heart failure) and the odds of losing glycemic control (HbA1c >7.0% after 52 weeks of therapy) were determined.* Results.* Bromocriptine-QR reduced the CVD endpoint by 48% (intention-to-treat; HR: 0.52 [0.28−0.98]) and 52% (on-treatment analysis; HR: 0.48 [0.24−0.95]). Bromocriptine-QR also reduced the odds of both losing glycemic control (OR: 0.63 (0.47−0.85), *p* = 0.002) and requiring treatment intensification to maintain HbA1c ≤7.0% (OR: 0.46 (0.31−0.69), *p* = 0.0002).* Conclusions. * Bromocriptine-QR therapy slowed the progression of CVD and metabolic disease in T2DM subjects in good glycemic control.

## 1. Introduction

Cardiovascular disease (CVD) is the leading cause of death among patients with type 2 diabetes mellitus (T2DM) even with earlier detection and treatment of T2DM as has occurred during the past decade. This patient population has a 2- to 3-fold higher risk of CVD relative to their nondiabetic counterparts [1]. Yet, the relationship between plasma glucose concentration and CVD in T2DM remains poorly understood.

Several in vitro and in vivo studies have documented that hyperglycemia alters cellular biochemistry within the vasculature, ultimately inducing endothelial dysfunction and abnormalities in vascular structure and biology predisposing T2DM patients to CVD (reviewed in [2]). Nonetheless, large randomized clinical studies evaluating the impact of improving glycemic control on CVD outcomes [3–6] failed to demonstrate an effect of improvement in existing hyperglycemia from HbA1c levels >7.0 to ≤7.0 to reduce CVD outcomes over the duration of the trials. However, posttrial follow-up and post hoc analyses of some of these trials suggest that early reductions in HbA1c may be coupled to modest long-term improvements in CVD outcomes [7, 8]. In addition, epidemiological studies indicate that the risks of vascular complications are strongly associated with glycemic exposure, such that the CVD event rate increases over time as the mean HbA1c increases above 7.0% prior to the first event [9–14]. In aggregate, these studies suggest that the effect of reducing existing hyperglycemia to reduce CVD event rate may require a long time to be expressed and is of moderate impact [15]. Apparently, progression of T2DM, with its multifactorial pathological components of the insulin resistance syndrome, including loss of glycemic control, predisposes to increased incidence of CVD that cannot then be easily, quickly, and largely reversed by merely reversing the hyperglycemia. Moreover, even in T2DM subjects with good glycemic control, increased risk of CVD is evident. Clearly, more effective approaches outside of managing dysglycemia are required to ameliorate these macrovascular complications of T2DM. In this regard, several lines of evidence suggest that other mechanisms such as increased vascular sympathetic tone and/or endothelial dysfunction may be strong potentiators of CVD in insulin resistant states [16–21]. Elevated sympathetic tone contributes significantly to hypertension, cardiac autonomic neuropathy, insulin resistance, dyslipidemia, vascular reactive oxygen species generation, inflammation, and endothelial dysfunction that each in turn contributes to CVD [16–21].

A current unmet goal of clinical research and practice in T2DM is the identification of safe and effective therapies that maintain good glycemic control, preventing progression of disease, while also reducing the long-term CVD risk, independent of their impact upon glycemic control. Bromocriptine-QR, a quick release formulation of bromocriptine, a sympatholytic dopamine D2 receptor agonist [22, 23] (approved by the U.S. Food and Drug Administration for the treatment of hyperglycemia in patients with T2DM in 2009) may offer a therapy with the potential to maintain glycemic control and reduce CVD risk [24–28]. Once daily, morning administration of bromocriptine-QR has been shown to improve glycemic control when used as either monotherapy or add-on therapy (0.5 to 0.9 HbA1c reduction relative to placebo control) in T2DM subjects with poor glycemic control (HbA1c ≥ 7.5) [27, 28]. In addition, in a large T2DM study population (Cycloset Safety Trial (CST); *N* = 3070) comprised of subjects across a wide range of glycemic control status (A1c range: 5.5–10.5; median [25th–75th percentile]: 6.8 [6.2–7.6]) whose hyperlipidemia and hypertension were well controlled and yet with preexisting CVD history (33% of population), intervention with this agent resulted in a 40% hazard risk reduction of a prespecified composite CVD endpoint over a period of one year [24]. Available evidence suggests that bromocriptine-QR may work through restoration of the daily morning peak in central circadian dopaminergic neural activities [29–32] to reduce the major CVD risk factors of hyperactive sympathetic tone at the vasculature, endothelial dysfunction, and vascular oxidative and nitrosative free radical generation [33, 34], each independently of any effect on fasting plasma glucose or lipid levels. These neuroendocrine aberrations are operative in progression of CVD over the entire continuum of glycemic control in T2DM patients, including those with good glycemic control, as they are also present in the prediabetic state [35–38]. Therefore, we hypothesized (1) that even in T2DM subjects with good glycemic (and lipid and blood pressure) control with standard of care, the CVD event rate may still be high due to the presence of these underlying etiological neuroendocrine pathologies and (2) that such subjects would benefit from administration of bromocriptine-QR to produce a potent and rapid reduction in CVD event rate via its beneficial effects on the above neuroendocrine pathologies, irrespective of such existing good glycemic (lipid and blood pressure) control. However, the antidiabetes and CVD protective effects of bromocriptine-QR in this specific cohort of T2DM subjects with well-controlled glycemia have never been investigated. Hence, to test this hypothesis, we analyzed the data from the Cycloset Safety Trial (CST) [24], which had enrolled subjects across a wide spectrum of glycemic control status ranging from very good to very poor control and afforded the unique opportunity to investigate, for the first time, the effects of bromocriptine-QR on progression of both dysglycemia and CVD in a relatively large population of subjects with well-controlled T2DM (HbA1c ≤ 7.0; median [25th–75th percentile]: 6.4 [6–6.7] in contradistinction to CST subjects with HbA1c >7.0; median [25th–75th percentile]: 7.8 [7.4–8.5]).

## 2. Methods

### 2.1. Study Subjects and Design

The current study population was derived from those subjects within the CST [24] with a baseline HbA1c ≤7.0. Of the 3070 subjects randomized 2 : 1 to treatment with bromocriptine-QR versus placebo in CST, 1834 subjects (1219 receiving bromocriptine-QR and 615 receiving placebo) had baseline HbA1c ≤7.0. The study protocol and design for the CST have been previously described [24]. Briefly, this was a 12-month, multicenter, placebo-controlled, double-blind, parallel-group safety and efficacy study in outpatient T2DM subjects recruited from general practice and diabetes clinics across 74 clinical centers in the United States and Puerto Rico. Subjects were between the ages of 30 and 80 years and had a body mass index <43 kg/m^2^ and an HbA1c ≤10.0%. Subjects with New York Heart Classifications I and II congestive heart failure (CHF) were allowed to participate, as were subjects with a history of myocardial infarction (MI) or coronary revascularization occurring >6 months before enrollment. Subjects were required to have maintained a stable diabetes treatment regimen for ≥30 days prior to randomization, consisting of either lifestyle interventions of medical nutrition therapy and appropriately prescribed physical activity, oral antihyperglycemic agents (≤2), or insulin either alone or in combination with 1 oral antihyperglycemic agent.

The study drug was titrated by adding 1 tablet (0.8 mg bromocriptine-QR per tablet) per week until a maximum tolerated daily dose between 2 and 6 tablets (1.6 to 4.8 mg/day) was achieved. The study drug was taken once daily with the morning meal, within 2 hours of waking. Subjects were required to continue their established antihyperglycemic treatments during the first 3 months of the study. However, the dosages of the oral agents or insulin could be modified as deemed appropriate by the study site investigator. After 3 months, alterations in the diabetes treatment regimen were allowed, if deemed necessary by the study site investigator, as long as these changes did not result in a final regimen that exceeded 2 oral agents or insulin plus 1 oral agent, exclusive of the study drug.

The study protocol was approved by site-specific or central institutional review boards and all subjects provided written informed consent to participate in the study before enrollment. This current study and analyses are original and different from any previously reported results from the Cycloset Safety Trial.

### 2.2. Study Endpoints and Statistical Analyses

#### 2.2.1. Two Primary Endpoints Were Evaluated in This Study

*CVD Endpoint*. The same prespecified CVD endpoint of the CST (a composite of major cardiovascular events, defined as a composite of first MI, stroke, coronary revascularization, or hospitalization for angina or CHF that occurred after randomization) was also used in this new study and analysis. An independent event adjudication committee consisting of two cardiologists and an endocrinologist, blinded to treatment assignment and Medical Dictionary for Regulatory Activities coding of events by the study team, made the final serious adverse event (SAE) system organ class (SOC) classifications and assignment of an SAE as a CVD endpoint. Statistical analyses were performed using Cox proportional-hazards regression. Intention-to-treat (ITT) and on-treatment (OT) analyses were conducted. ITT analysis included all patients receiving at least one dose of the study drug with exposure time being one year or time to event. To account for any possible influence of weighted early termination among bromocriptine-QR versus placebo treated subjects that might artificially impact the ITT analysis, an OT analysis restricting exposure time to time on study drug was also conducted. Superiority between bromocriptine-QR and placebo for the CVD endpoint was defined as the upper bound of the two-sided 95% confidence limit being <1.0 and the superiority analysis of CVD endpoint was based on the Cox proportional-hazards regression, adjusted for baseline covariates including history of stroke, revascularization, and center, with two-sided *p* values calculated. The cumulative incidence rate of the CVD endpoint was analyzed by log-rank test. A Kaplan-Meier curve for events over time was generated. The significance level was set at *p* < 0.05.

*Glycemic Control Endpoint.* The impact of bromocriptine-QR versus placebo on the progression of diabetes was described as the odds of losing glycemic control while on therapy and determined by evaluating the percent of subjects completing 52 weeks of therapy, whose HbA1c progressed above 7.0%. The odds of requiring treatment intensification to maintain HbA1c ≤7.0% during the study period of 52 weeks were also analyzed. To eliminate potential confounding arising from intensification of concomitant antidiabetes medications, the analyses were also performed in only those subjects completing 52 weeks without requiring any concomitant antidiabetes treatment intensification. The above analytical approach was also conducted on the entire study population using a last observation carry-forward analysis.

Statistical analyses of the primary endpoints and safety measures were conducted independently by Everest Inc. (Ontario, Canada) using SAS software version 8.2 (Cary, NC).

Safety analyses were conducted as previously described for the CST [24].

## 3. Results

### 3.1. Baseline Characteristics of Study Subjects

There were no significant differences in baseline demographics between the bromocriptine-QR and placebo study arms although the HbA1c level was not considered as a stratification factor in the CST (Table 1(a)). The study population carried multiple risk factors for CVD and was comprised of individuals among whom 74% were hypertensive, 75% were hyperlipidemic, greater than 50% were prior or current smokers, approximately 33% had preexisting CVD who were obese on population average with an average BMI of >32 (Table 1(a)). Fasting plasma glucose, lipids, and blood pressure were well controlled in both groups. Majority of the subjects were receiving cardioprotective medications (see Table 1(b) for details).

### 3.2. Subject Disposition

**Figure 1:**
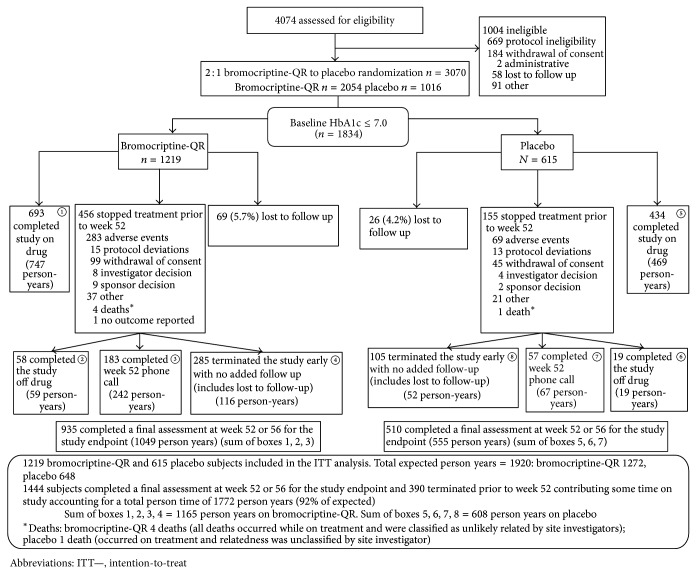
Disposition of study subjects.

1834 patients (1219 receiving bromocriptine-QR and 615 receiving placebo) from the CST trial had baseline HbA1c ≤7.0% and were included in this study. In total, 92% of the planned person-year CV outcome ascertainment was observed in this trial (1772 of 1920 possible total person-years), with 77% of bromocriptine-QR subjects and 83% of placebo subjects providing a week 52 plus 30-day follow-up outcome assessment. The number of subjects with HbA1c assessment at Week 52 was 1203 (750 bromocriptine-QR and 453 placebo). Details of subject disposition and delineation of person-year CV outcome ascertainment are shown in Figure 1.

### 3.3. CVD Endpoint

**Figure 2:**
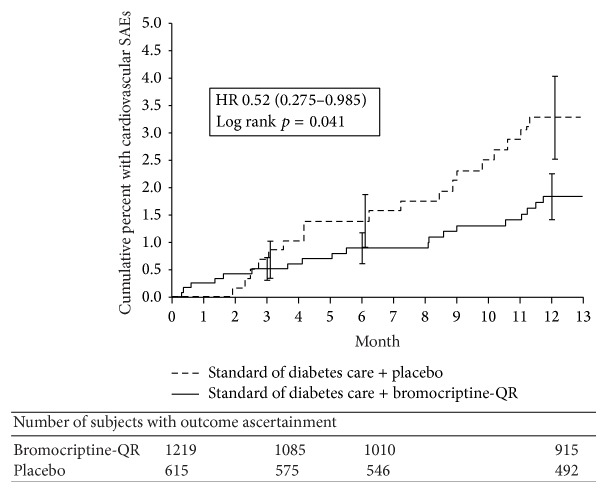
Kaplan-Meier estimates of the proportion of subjects by treatment that experienced an event within the composite CVD endpoint.

In the ITT analysis, the composite CVD endpoint occurred in 19 bromocriptine-QR-treated (1.6%) and 19 placebo-treated (3.1%) subjects, resulting in a 48% CVD hazard risk reduction (hazard ratio [HR] 0.52, CI 0.28−0.98) (Table 2). The OT analysis revealed a 52% CVD risk reduction (HR: 0.48; CI: 0.24−0.95) (Table 2).  Figure 2 depicts the Kaplan-Meier curve of the cumulative incidence rate of the composite CVD endpoint by treatment and demonstrates a significant difference at 1 year (log rank *p* = 0.041).

There were no significant changes in plasma lipid levels or heart rate in either treatment group. Compared to placebo, the bromocriptine-QR treated group exhibited a mild reduction in blood pressure (change from baseline blood pressure (mean ± SD; mmHg): −2.81 ± 16.27 systolic and −1.89 ± 9.76 diastolic in the bromocriptine group; −0.54 ± 14.86 systolic and −0.56 ± 9.54 diastolic in the placebo group; between group difference (CI): −1.76 (−3.09, −0.42) systolic (*p* = 0.0099) and −1.19 (−2.00, −0.38), (*p* = 0.0038) diastolic).

### 3.4. Glycemic Control Endpoint

Among study subjects whose HbA1c remained ≤7.0 during the study period, bromocriptine-QR intervention reduced the fraction of subjects that required intensified concomitant antidiabetes therapy to do so by 47%for the completer population and by 58% for the ITT population (see Table 3 for details).

Analyzing all subjects who had a 52-week HbA1c measurement, irrespective of changes in concomitant diabetes therapy and adjusting for baseline HbA1c, the odds of losing glycemic control (HbA1c >7.0% after 52 weeks of treatment) were significantly lower with bromocriptine-QR therapy (OR (95% CI): 0.63 (0.47–0.85), *p* = 0.002). The odds of requiring treatment intensification to maintain HbA1c ≤7.0% over the course of the study were also significantly lower with bromocriptine-QR therapy (OR (95% CI): 0.46 (0.31–0.69), *p* = 0.0002). On repeating the analysis including only those subjects who did not have a change in the intensity of their concomitant diabetes regimen, bromocriptine-QR was still associated with significantly lower odds of losing glycemic control compared with placebo (18% versus 26%, resp.) (OR (95% CI): 0.56 (0.39–0.80), *p* = 0.002). These differences between the treatment groups were unaffected and remained significant (*p* = 0.001) after adjusting for body weight changes during the study. The same results described above were observed using a last observation carry forward analysis among all subjects with a screening HbA1c value ≤7.0% (ITT population, *N* = 1834) (see Table 3 for details). For the ITT population, the between-group difference in change from baseline HbA1c (6.3 ± 0.5) was −0.17 (CI −0.23, −0.11; bromocriptine-QR: 0.18, placebo: 0.35; *p* < 0.0001).

### 3.5. Safety Analyses

**Figure 3:**
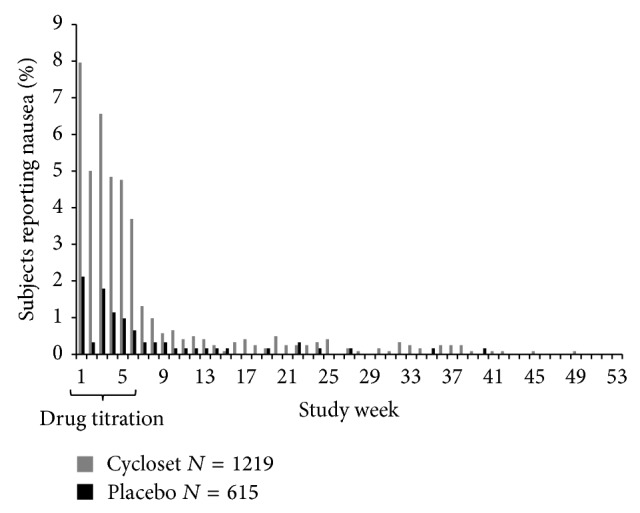
Occurrence of nausea (most commonly reported adverse event) by study week.

Adverse events (AEs) that were most commonly reported (occurring in greater than 5% of subjects in either group) are shown in Table 4 and were similar to those previously reported for the CST. Among the AEs occurring at a higher rate in the bromocriptine-QR group, the between-group difference was significant (*p* < 0.0001) for nausea (31.7% versus 8.0%), dizziness (15.5% versus 8.6%), fatigue (13.9% versus 7%), and vomiting (8.7% versus 3.4%), with the severity reported as being mild-moderate in >90% of all cases in each of these categories. The increased rate of nausea, the most common adverse event reported, was transient and confined to the initial 6-week drug titration period with an average weekly rate of approximately 6% and decreasing to <1% thereafter (see Figure 3). Hypoglycemic episodes occurred infrequently (5.5% bromocriptine-QR versus 4.2% placebo) with no significant between-group difference in the rate. Arthralgia was reduced by 39% in the bromocriptine-QR group (*p* = 0.03).

In the bromocriptine-QR treated group 105 subjects (8.6%) reported 149 serious adverse events (SAE) while the placebo-treated group had 58 subjects (9.4%) reporting 80 serious adverse events. In the cardiac disorders body system class there were 33 events reported in 28 subjects (2.3%) in the bromocriptine-QR group and 25 events reported in 21 subjects (3.4%) in the placebo group. No other body system classes had SAE occurring in greater than 2% of either group.

There was no between group difference in change from baseline body weight (bromocriptine-QR: 0.386 versus placebo: 0.366, *p* = 0.97).

## 4. Discussion

The findings of this study demonstrated that, in metabolically well-controlled T2DM subjects (baseline HbA1c ≤7.0%), adding bromocriptine-QR treatment to their baseline established diabetes treatment regimen significantly aided in maintaining good glycemic control, with a lower likelihood of progressing above an HbA1c of 7.0% or requiring intensification of treatment over the ensuing year to maintain HbA1c ≤7.0%. Furthermore, bromocriptine-QR treatment was also associated with a 48% CVD event rate reduction within the 1-year study duration in this population of T2DM subjects, with excellent baseline glycemic control (HbA1c 6.3 ± 0.5). The study population had multiple cardiometabolic risk factors for cardiovascular disease at baseline (Table 1(a)) and the placebo-arm of the study population had a relatively high event rate (3.1%) for the prespecified serious cardiovascular adverse event endpoint while being well controlled pharmacologically for dysglycemia and on population average for dyslipidemia and hypertension as well. These findings suggest that, in this T2DM subject population demographic, (1) there are biochemical pathological factors beyond hyperglycemia, hyperlipidemia, and high blood pressure predisposing this population to CVD events and (2) there likely are significant mechanisms beyond glycemic control contributing to the observed CVD risk reduction with this therapy.

Although epidemiological evidence [9–14] supports an adverse role of poor glucose control on CVD risk, intervention trials have been less conclusive. Of the earlier studies, the United Kingdom Prospective Diabetes Study (UKPDS) demonstrated that intensive glycemic control in individuals with newly diagnosed T2DM reduced the risk of microvascular complications [3]. Further, longer-term follow-up of these individuals for 10 more years after the end of the intervention trial demonstrated continued reductions in microvascular disease risk and statistically significant benefits on both CVD endpoints and total mortality in the intensive therapy arm, despite the mean HbA1c between the groups having converged soon after the randomized phase of the trial had concluded [7]. The Action to Control Cardiovascular Risk in Diabetes (ACCORD) study [4], the Action in Diabetes and Vascular Disease (ADVANCE) study [5], and the Veterans Affairs Diabetes Trial (VADT) [6] that were shorter in duration than UKPDS enrolled older patients, with more advanced and poorly controlled diabetes, and known CAD or at high risk for CVD and found no significant decrease in CVD endpoints with intensive glucose control. However, the evidence from/post hoc analyses of these large trials [8–10] and the longer-term UKPDS follow-up [7] suggests long-lasting benefits of tighter glycemic control in patients that are younger and earlier in the course of their diabetes or with lower HbA1c values (at about 7.0) at treatment intensification. In these individuals, maintaining HbA1c <7.0% remains a reasonable target and may have important benefits in reducing the future burden of macrovascular and microvascular disease. The bromocriptine-QR effect to slow progression of dysglycemia in these individuals as demonstrated herein may therefore offer CVD benefits over the long-term; however, the rapid response to bromocriptine-QR respecting the reduction in CVD outcomes observed in the present study begs for other mechanisms operative in the manifestation of this effect. Moreover, although bromocriptine has been shown to reduce hypertriglyceridemia [39] and elevated blood pressure [23], these effects cannot be responsible for the observed CVD event rate reduction as these parameters were well controlled at baseline and minimally affected by the intervention. Prior studies of the neuroendocrine impact of timed bromocriptine administration in insulin resistant animals and humans however may offer insights into a possible mechanism for the observed CVD (and metabolic) response to the therapy as follows.

Bromocriptine-QR therapy is a circadian-timed administration of a quick-release, high absorbing, and short half-life formulation of bromocriptine. It has been formulated and administered in the morning within 2 hours of waking to provide a discrete and brief daily interval of circulating bromocriptine [24–28, 39], thereby providing a timed pulse of increased dopaminergic activity centrally at the time of day that studies suggest is the natural daily peak of central dopaminergic activity in healthy individuals [32, 40]. Studies indicate that disturbed circadian rhythmicity of the biological clock (hypothalamic suprachiasmatic nucleus (SCN)) and a reduced dopaminergic tone within the central nervous system are associated with the development of insulin resistance, obesity, and diabetes [26, 29, 32, 35, 41–47]. A diminution of the daily circadian peak in dopaminergic activity at the SCN at the onset of the daily locomotor activity rhythm (e.g., waking from night-time sleep in humans) is coupled to increases in hypothalamic ventromedial and paraventricular nuclei drive for increased sympathetic and hypothalamic-pituitary-adrenal (HPA) axis activities (reviewed in [35]). These increased sympathetic/HPA activities potentiate increases in adipose lipolysis and subsequent plasma free fatty acid levels, increases in hepatic glucose and lipid output and decreases in hepatic glucose storage, and increased peripheral insulin resistance [33, 35], particularly during the postprandial state [36]. When bromocriptine is administered at the appropriate time of day to restore normal SCN dopaminergic activity in insulin resistant states, it normalizes such aberrant hypothalamic functions and elevated sympathetic tone and the HPA axis circadian activity [35]. When administered to humans in the early morning upon waking in an effort to restore the normal waking rise in central dopaminergic activity that is diminished in insulin resistant states [32, 35], bromocriptine-QR improves insulin resistance and other metabolic abnormalities [26–28, 39, 42].

While the mechanisms by which timed bromocriptine-QR therapy produces the observed effects on CVD outcomes are yet to be fully delineated, available evidence suggests important CVD-protective roles for its modulation of central nervous system and circadian hypothalamic functions to reduce elevated sympathetic nervous system (SNS) (and HPA axis) activities (as described above) that directly and indirectly potentiate vascular inflammation, endothelial dysfunction, and arterial stiffening [16–18] and that are coupled to increased CVD risk if overactive [16, 48]. Such SNS influences on adipose and liver potentiate their increased secretion of FFA/lipid and inflammatory cytokines that in turn cause vascular inflammation and reactive oxygen species generation that are damaging to the vasculature [19–21]. Increases in SNS activities can also induce a proinflammatory response in various arms of the immune system itself that can also predispose to vascular damage [49]. Additionally, and likely much more importantly, increased SNS activity can produce adverse cardiometabolic effects directly upon the vasculature to potentiate vasoconstriction, generation of vascular reactive oxygen and nitrogen species, increased inflammation, endothelial dysfunction, and arteriosclerosis [16–18, 50]. Furthermore, changes in vascular inflammation and endothelial function (positive or negative) can manifest changes in vascular pathology/physiology quickly [16–21, 37, 38]. Importantly, circadian timed treatment of spontaneously hypertensive rats (SHR) with bromocriptine has demonstrated improvements in metabolic syndrome parameters including elevated SNS tone, fatty liver, and hepatic inflammation [33], as well as reductions in arterial stiffness and endothelial nitric oxide synthase uncoupling [33], two phenomena commonly observed in patients with T2DM and strongly linked to progression of macrovascular disease [19–21]. Others have reported similar hepatic and vascular findings in response to bromocriptine therapy in other animal models [51, 52]. Also, beyond normalizing (resetting) hypothalamic control of elevated sympathetic tone and vascular dysfunction, bromocriptine has direct sympatholytic activity due to its neurotransmitter receptor modulation capacity [22, 23, 53]. Endothelial dysfunction, vascular inflammation, and elevated sympathetic tone are early pathological events in the progression of CVD and precede the onset of T2DM [16–21, 37, 38]. As such, these pathologies may well have contributed to the 3.1% CVD event rate observed in this study placebo population that was well controlled for hyperglycemia, hyperlipidemia, and high blood pressure with standard of care therapy. That is to say, such T2DM subjects whose dysglycemia, dyslipidemia, and hypertension are well controlled with standard of care pharmacotherapy still remain in need of therapy for their underlying substantive CVD risk due to these above described neuroendocrine pathologies. Therefore, if these pathologies are the targets of this bromocriptine-QR therapy, as available evidence suggests, then the present findings suggest that early intervention with this therapy in the course of T2DM may potentially provide longer-term benefit of CVD risk reduction.

The limitations of this study include the relatively small number of CVD events and the short duration of the trial. Other limitations include the lack of mechanistic information relating to bromocriptine-QR impact on sympathetic tone or endothelial dysfunction in the study population. Therefore, caution should be exercised when evaluating these CVD findings and their full potential clinical ramifications. The abovementioned mechanisms proposed to be operative in the observed CVD outcome results of this study need to be further investigated in human studies (e.g., impact of bromocriptine-QR on vascular inflammation, endothelial dysfunction, vascular and systemic reactive oxygen species status, and aortic compliance) before definitive conclusions on such mechanisms can be made. It should be appreciated though that the study subjects had multiple risk factors for CVD at baseline and the prespecified CVD endpoint occurred in the placebo arm at a relatively high rate despite well-controlled hyperglycemia, hyperlipidemia, and blood pressure and that the reduction in CVD events with bromocriptine-QR intervention was evident within the short duration of this study in such a population of T2DM subjects. Similarly, in this regard, it should be noted that the incidence rate for the prespecified CVD endpoint among subjects in the CST with baseline HbA1c >7.0% was 16/830 (1.9%) and 12/400 (3.0%) in the bromocriptine-QR and placebo arms, respectively, comprising approximately half of the total events in the original CST and yielding a hazard ratio of 0.74 (CI: 0.35–1.56) for this subset (HbA1c >7.0%). Although this reduction in CVD events in this subset was not statistically significant likely due to the small *N* number, these findings suggest an operative impact of this therapy in CVD risk reduction across the continuum of glycemic control status. CVD is the leading cause of death in T2DM subjects and yet there are currently no antidiabetes medications available with proven cardioprotective benefits. Consequently, the CVD outcome findings described herein along with those from other bromocriptine-QR intervention studies of T2DM subjects [24, 25] suggest that bromocriptine-QR should be considered for further investigation in larger, longer term studies to establish or not the validity of its potential use early in the course of T2DM as an cardioprotective agent.

## 5. Conclusion

In conclusion, the findings of this study support that bromocriptine-QR therapy among T2DM subjects in good glycemic control (HbA1c ≤7.0) reduced progression of dysglycemia and reduces CVD event rate within one year of therapy. Reducing CVD remains a major unmet medical need in T2DM. While reducing hyperglycemia may contribute to such an outcome, the overall effects are modest. Other major vascular risk factors such as vascular sympathetic tone and endothelial dysfunction are present early in T2DM disease progression (e.g., obesity/prediabetes) and represent important therapeutic targets for CVD event rate reduction even in subjects with good glycemic and metabolic control. Bromocriptine-QR is a sympatholytic dopamine D2 receptor agonist that appears to reduce these vascular risk factors via the neuroendocrine axis and reduce progression of CVD in T2DM even in the setting of good glycemic control. The present findings suggest that further larger, longer term studies to assess the value of early intervention with bromocriptine-QR during the chronology of T2DM to provide unique long-term cardiovascular health benefits are warranted.

## Figures and Tables

**Table:** (a)

Variable	All patients with incoming HbA1c ≤7.0		Patients with incoming A1c ≤7.0 that completed 52 study weeks	
	Bromocriptine-QR (*N* = 1,219)	Placebo (*N* = 615)	Bromocriptine-QR (*N* = 750)	Placebo (*N* = 453)
Age (years)	60.4 ± 10.1	60.4 ± 10.1	60.9 ± 9.6	60.8 ± 9.9
Duration of diabetes (years)	6.8 ± 6.9	6.6 ± 6.5	6.7 ± 6.5	6.7 ± 6.8
Male sex	673 (55)	335 (54)	454 (61)	262 (58)
Race:				
White	869 (71)	431 (70)	546 (73)	327 (72)
Black	183 (15)	110 (18)	113 (15)	76 (17)
Hispanic	142 (12)	63 (10)	74 (10)	41 (9)
Asian	10 (1)	6 (1)	7 (1)	5 (1)
Other	15 (1)	5 (1)	10 (1)	4 (1)
Comorbid conditions				
Hypertension	908 (74)	457 (74)	558 (74)	348 (77)
Angina pectoris	119 (10)	57 (9)	79 (11)	45 (10)
Myocardial infarction	98 (8)	52 (8)	62 (8)	44 (10)
Revascularization	111 (9)	69 (11)	69 (9)	57 (13)
Stroke	43 (4)	29 (5)	29 (4)	21 (5)
Hypercholesterolemia^∗^	914 (75)	458 (74)	574 (77)	351 (77)
Hypertriglyceridemia^∗^	470 (39)	241 (39)	297 (40)	181 (40)
Current smoker	161 (13)	73 (12)	98 (13)	48 (11)
Former smoker	495 (41)	258 (42)	321 (43)	193 (43)
HbA1c (%)	6.3 ± 0.49	6.3 ± 0.48	6.3 ± 0.48	6.3 ± 0.47
Fasting glucose (mmol/L)	7.00 ± 1.56	6.89 ± 1.44	7.06 ± 1.56	6.83 ± 1.44
Total cholesterol (mmol/L)	4.58 ± 1.03	4.53 ± 0.93	4.47 ± 0.96	4.53 ± 0.93
LDL cholesterol (mmol/L)	2.51 ± 0.83	2.48 ± 0.75	2.43 ± 0.78	2.48 ± 0.75
HDL cholesterol (mmol/L)	1.19 ± 0.31	1.22 ± 0.31	1.19 ± 0.31	1.19 ± 0.28
Triglycerides (mmol/L)	1.94 ± 1.33	1.86 ± 1.22	1.93 ± 1.23	1.91 ± 1.32
Systolic BP (mmHg)	130 ± 14	129 ± 13	130 ± 14	129 ± 13
Diastolic BP (mmHg),	77 ± 9	77 ± 9	77 ± 9	76 ± 9
Creatinine (*μ*mol/L)	97.24 ± 17.68	97.24 ± 17.68	97.24 ± 17.68	97.24 ± 17.68
Body mass index (kg/m^2^)	32.2 ± 5.0	32.3 ± 5.1	32.3 ± 5.0	32.2 ± 5.0

Data are shown as means ± SD for continuous variables and number (%) for categorical variables.

^∗^Based on history as assessed by study site investigator.

**Table:** (b)

Variable	All patients with incoming HbA1c ≤7.0		Patients with incoming HbA1c ≤7.0 that completed 52 weeks of study	
	Bromocriptine-QR (*N* = 1,219)	Placebo (*N* = 615)	Bromocriptine-QR (*N* = 750)	Placebo (*N* = 453)
Diabetes Treatment Regimen				
Diet only	194 (16)	93 (15)	115 (15)	67 (15)
One oral hypoglycemic agent	570 (47)	304 (49)	365 (49)	221 (49)
Two oral hypoglycemic agents	350 (29)	154 (25)	203 (27)	117 (26)
Oral agent plus insulin	59 (5)	30 (5)	43 (6)	24 (5)
Insulin only	45 (4)	34 (6)	24 (3)	24 (5)
Not reported	1	0	0	0
Anti-diabetes Medications by Agent—no. (%)				
Insulin	104 (9)	64 (10)	67 (9)	48 (11)
Metformin	698 (57)	347 (56)	412 (55)	262 (58)
Thiazolidinediones	238 (20)	116 (19)	148 (20)	83 (18)
Sulfonylureas	406 (33)	187 (30)	260 (35)	141 (31)
Other	16 (1)	10 (2)	11 (1)	7 (2)

Cardio-protective Medications by Class—no. (%)				
ACE Inhibitors	578 (47)	275 (45)	370 (49)	216 (48)
Angiotensin II Receptor Inhibitors	215 (18)	123 (20)	127 (17)	92 (20)
Beta Blockers	278 (23)	156 (25)	180 (24)	125 (28)
Diuretics	411 (34)	215 (35)	255 (34)	158 (35)
Calcium Channel Blockers^b^	198 (16)	120 (20)	119 (16)	89 (20)
HMG CoA Reductase Inhibitor	711 (58)	360 (59)	452 (60)	274 (60)
Fibrate	86 (7)	42 (7)	59 (8)	36 (8)
Platelet Aggregation Inhibitors	554 (45)	288 (47)	363 (48)	221 (49)
Cardio-protective Medications by Number—no. (%)				
Taking 1 cardioprotective agent	218 (18)	101 (16)	127 (17)	78 (17)
Taking 2 cardioprotective agents	266 (22)	148 (24)	169 (23)	106 (23)
Taking 3 cardioprotective agents	238 (20)	113 (18)	157 (21)	88 (19)
Taking ≥4 cardioprotective agents	369 (30)	186 (30)	233 (31)	143 (32)

^a^Includes fixed dose combinations.

^b^Calcium channel blockers include dihydropryidine, pheny-alkylamine, benozothiazepine.

**Table 2:** Impact of bromocriptine-QR on a prespecified, adjudicated composite CVD endpoint, and individual components of the composite.

	Bromocriptine-QR *N* = 1219	Placebo *N* = 615	Hazard ratio (95% CI)
	# subjects (%)^a^	# subjects (%)^a^	
*Intention to treat analysis *			
Prespecified adjudicated composite CVD endpoint (ITT)	19 (1.6)	19 (3.1)	0.52 (0.28–0.98)
Composite CVD endpoint by each component			
Myocardial infarction	5 (0.4)	5 (0.8)	0.54 (0.16–1.86)
Stroke	1 (0.1)	3 (0.5)	0.18 (0.02–1.71)
Hospitalization for angina	4 (0.3)	3 (0.5)	0.71 (0.16–3.15)
Hospitalization for heart failure	3 (0.2)	4 (0.7)	0.36 (0.08–1.62)
Coronary revascularization	6 (0.5)	4 (0.7)	0.81 (0.23–2.86)
Coronary revascularization following a primary endpoint (e.g., CABG after MI)	7 (0.6)	7 (1.1)	0.53 (0.19–1.52)
*On treatment analysis *			
Prespecified adjudicated composite CVD endpoint	15 (1.2)	18 (2.9)	0.48 (0.24–0.95)

^a^% of events per total *N* per group (1219 bromocriptine-QR, 615 placebo).

CI: confidence interval, CV: cardiovascular, CABG: coronary artery bypass graft, MI: myocardial infarction.

**Table 3:** Effect of bromocriptine-QR versus placebo on odds of losing glycemic control (HbA1C going above 7.0) and odds of requiring intensification of concomitant diabetes treatment regimen to maintain HbA1C ≤7.0.

	ITT: last observation carried forward (LOCF) analysis			Week 52 completer analysis		
Effect of B-QR versus placebo on odds of losing good glycemic control (HbA1c exceeding 7.0) at study completion						

Study Group	% subjects with HbA1c exceeding 7.0		Odds Ratio^a^ (CI), *p* value	% Subjects with HbA1c exceeding 7.0		Odds Ratio^a^ (CI), *p* value
	Placebo	B-QR		Placebo	B-QR	

All subjects^b^ (baseline HbA1C ≤7.0)	24.7	15.2	0.505 (0.390, 0.653), *p* < 0.0001	28.5	21.1	0.632 (0.471, 0.849), *p* = 0.002
	B-QR versus P Δ = −38.5%		B-QR versus P Δ = −26.0%	

Subset of all subjects with no change in concomitant diabetes regimen during the study period^c^	22.1	13.2	0.469 (0.341, 0.644), *p* < 0.0001	25.6	17.7	0.558 (0.389, 0.800), *p* = 0.002
	B-QR versus P Δ = −40.3%		B-QR versus P Δ = −30.9%	

Effect of B-QR versus placebo on odds of requiring intensification of concomitant diabetes treatment regimen to maintain HbA1C ≤7.0						

Study Group	% Subjects intensifying regimen		Odds Ratio^a^ (CI), *p* value	% Subjects intensifying regimen		Odds Ratio^∗^ (CI), *p* value
	Placebo	B-QR		Placebo	B-QR	

Subset of all subjects that stayed in good glycemic control (HbA1c ≤ 7) during the study period^d^	15.6	6.6	0.365 (0.256, 0.521), *p* < 0.0001	17.9	9.5	0.463 (0.310, 0.690), *p* = 0.0002
	B-QR versus P Δ = −57.7%		B-QR versus P Δ = −46.9%	

Abbreviations: B-QR: bromocriptine-QR; P: placebo; CI: confidence interval.

^a^Adjusted for baseline HbA1c.

^b^Completer: *N* = 1203 (B-QR: 750; placebo: 453); LOCF: *N* = 1834 (B-QR: 1219; placebo: 615).

^c^Completer: *N* = 914 (B-QR: 586; placebo: 328); LOCF: *N* = 1358 (B-QR: 924; placebo: 434).

^d^Completer: *N* = 916 (B-QR: 592; placebo: 324); LOCF: *N* = 1497 (B-QR: 1034; placebo: 463).

**Table 4:** Most commonly reported (≥5% in either treatment group) adverse events.

	Bromocriptine-QR *N* = 1219	Placebo *N* = 615
	*N* (%)	*N* (%)
Nausea	386 (31.7)^∗^	49 (8.0)
Dizziness	189 (15.5)^∗^	53 (8.6)
Headache	141 (11.6)	54 (8.8)
Fatigue	170 (13.9)^∗^	43 (7.0)
Vomiting	106 (8.7)^∗^	21 (3.4)
Constipation	69 (5.7)	31 (5.0)
Hypoglycemia	67 (5.5)	26 (4.2)
Diarrhea	92 (7.5)	49 (8.0)
Nasopharyngitis	64 (5.3)	34 (5.5)
Upper respiratory infection	60 (4.9)	40 (6.5)
Arthralgia	47 (3.8)^†^	38 (6.2)

^∗^Between-group difference *p* < 0.0001.

^†^Between-group difference *p* = 0.03.
